# Evaluating a grading change at UCSD school of medicine: pass/fail grading is associated with decreased performance on preclinical exams but unchanged performance on USMLE step 1 scores

**DOI:** 10.1186/1472-6920-14-127

**Published:** 2014-06-30

**Authors:** Susan GR McDuff, DeForest McDuff, Jennifer A Farace, Carolyn J Kelly, Maria C Savoia, Jess Mandel

**Affiliations:** 1 University of California, San Diego School of Medicine, 9500 Gilman Drive #0606, San Diego, CA 92093-0606, USA; 2 Quant Economics, Inc., San Diego, CA 92130, USA

**Keywords:** Medical education, Pass-fail grading, Honors-pass-fail grading, Educational measurement

## Abstract

**Background:**

To assess the impact of a change in preclerkship grading system from Honors/Pass/Fail (H/P/F) to Pass/Fail (P/F) on University of California, San Diego (UCSD) medical students’ academic performance.

**Methods:**

Academic performance of students in the classes of 2011 and 2012 (*constant-grading classes*) were collected and compared with performance of students in the class of 2013 (*grading-change class*) because the grading policy at UCSD SOM was changed for the class of 2013, from H/P/F during the first year (MS1) to P/F during the second year (MS2). For all students, data consisted of test scores from required preclinical courses from MS1 and MS2 years, and USMLE Step 1 scores. Linear regression analysis controlled for other factors that could be predictive of student performance (i.e., MCAT scores, undergraduate GPA, age, gender, etc.) in order to isolate the effect of the changed grading policy on academic performance. The change in grading policy in the MS2 year only, without any corresponding changes to the medical curriculum, presents a unique natural experiment with which to cleanly evaluate the effect of P/F grading on performance outcomes.

**Results:**

After controlling for other factors, the grading policy change to P/F grading in the MS2 year had a negative impact on second-year grades relative to first-year grades (the constant-grading classes performed 1.65% points lower during their MS2 year compared to the MS1 year versus 3.25% points lower for the grading-change class, *p* < 0.0001), but had no observable impact on USMLE Step 1 scores.

**Conclusions:**

A change in grading from H/P/F grading to P/F grading was associated with decreased performance on preclinical examinations but no decrease in performance on the USMLE Step 1 examination. These results are discussed in the broader context of the multitude of factors that should be considered in assessing the merits of various grading systems, and ultimately the authors recommend the continuation of pass-fail grading at UCSD School of Medicine.

## Background

There is currently debate regarding the optimal grading system to evaluate medical student performance during the preclinical years. According to the most recent data available from the Association of American Medical Colleges, the majority of schools assign some form of honors designation to students for preclinical coursework (of 56 schools surveyed in 2011, 37 schools awarded honors and 19 schools did not) [1]. However, many medical schools have recently switched from a tiered grading system (e.g., letter grades, honors/pass/fail) to a pass/fail system [1], which makes it increasingly important to understand the merits and drawbacks of both systems.

Proponents of tiered grading systems believe that such systems afford predictive value in terms of identifying students who are in need of additional help [2]. For example, Gonnella et al. conducted a prospective, longitudinal study of 6656 Jefferson Medical College students [2]. The researchers grouped the students into 10 groups based upon their numeric grades (i.e., a 1–100 continuous scale) in the first year of medical school. They found a statistically significant association between performance in the first year of medical school and year 2 GPA, year 3 clinical examination grades, ratings of clinical competence in core clerkships, and medical school class rank [2]. The authors identified significant differences between the 10 groups in terms of performance on all three steps of the USMLE, and even on ratings of post-graduate clinical competence. Based on their results, Gonnella et al. argue that tiered grading systems are an important means of identifying students for whom additional instruction may be helpful, and that important information is lost when educators move from using an honors/pass/fail approach to a pass/fail approach.

Other advocates of tiered systems argue that students’ abilities to obtain desired residency programs may be hindered by attending schools with pass/fail grading systems [3]. For example, Spring et al. conducted a literature review of papers that examined the effect of pass/fail grading on academic and well-being outcomes [3]. While the authors concluded that objective academic performance is not adversely affected by a pass/fail grading system and that student well-being is enhanced, they noted that some studies suggested that residency program directors believe pass/fail grading creates disadvantages for students in the matching process.

Supporters of a pass/fail grading system argue that such systems improve students’ psychological well-being, decrease competitiveness, and promote cooperative learning while minimizing impact on academic performance [4,5]. For example, Bloodgood et al. investigated the effect of a change from letter grades to pass/fail grading on academic and psychological outcomes at the University of Virginia School of Medicine [4]. They found no reduction in performance in courses, clerkships, USMLE test scores, success in residency placement, or level of attendance in classes associated with the grading change; however, students rated themselves higher on subjective measures of psychological well-being and satisfaction [4]. Reed et al. surveyed students from 12 different medical schools and found that students attending schools with tiered grading systems had higher levels of stress, emotional exhaustion, depersonalization, and burnout compared with students graded using pass/fail [5]. Finally, White & Fantone studied outcomes associated with a switch from tiered to pass/fail grading and found that students graded with pass/fail performed worse in two preclinical courses, better in one course, and no different from students graded with a tiered system in terms of MCATs, GPAs, average performance in the rest of the second year courses, USMLE Step 1 scores, and residency placement [6]. While there appears to be consensus that pass/fail grading systems are associated with improved student well-being [4,5], the impact of such systems on academic outcomes is less clear [2,6].

In order to further explore academic outcomes associated with tiered and pass/fail grading, this paper presents educational outcomes associated with the grading policy change implemented at the UCSD School of Medicine during the 2010–2011 school year. Historically, the preclinical curriculum at UCSD used an Honors/Pass/Fail grading system. Beginning in the 2010–2011 academic year, the UCSD School of Medicine switched to using a Pass/Fail grading system for the preclinical curriculum, with no change in the content or structure of the curriculum at that time. This policy change provides a unique natural experiment to compare the preclinical course performance from the SOM classes of 2011 and 2012 (graded using H/P/F) with the performance from the SOM class of 2013 (graded using P/F) to assess whether the grading change impacted students’ performance in required preclinical coursework and the USMLE Step 1 exam.

## Methods

### Description of the UCSD School of Medicine curriculum and grading

This paper summarizes the results of a comparison of two groups of medical students at the University of California, San Diego School of Medicine: students with H/P/F grading regime (classes of 2011 and 2012, n = 243) and students with P/F grading (class of 2013, n = 117). Students in the first group underwent P/F grading during the first quarter of the first year of medical school (MS1 year), and were graded with H/P/F grading during the remainder of the MS1 and MS2 preclinical years (5 additional quarters). Students in the second group had the MS1 year graded the same as the classes before them (*i.e.*, the first quarter was graded P/F and the remaining 2 quarters were graded as H/P/F), but had the MS2 year graded P/F for all 3 quarters. Additional file 1: Table S1 presents a summary of the grading policy used in each quarter for the students involved in the analysis. Note that an academic year at UCSD is comprised of 4 quarters, although the summer quarter is ungraded during the preclinical curriculum. Therefore, the preclinical curriculum is comprised of 6 total quarters.

The curriculum (coursework and examinations) given during these years was essentially unchanged for both student groups and was unaffected by the comprehensive change in preclinical curriculum at UCSD that began with the class of 2014. In their MS1 year, all students took essentially the same complement of courses during the Fall Quarter (a. Cell Biology and Behavior, consisting of biochemistry, molecular biology, and immunology; b. Social and Behavioral Sciences: Doctor-Patient Relationship; and c. Principles of Pharmacology), Winter Quarter (a. Organ Physiology; and b. Principles of Pharmacology), and Spring Quarter (a. Basic Neurology; b. Principles of Pharmacology; c. Endocrinology and Reproductive Medicine; and d. Social and Behavioral Sciences: Human Growth and Development). However, for the classes of 2012 and 2013, a brief introduction to Genetics was offered in the Fall quarter that was not offered to the class of 2011. In their MS2 year, all students took the same complement of courses during the Fall Quarter (a. Hematology; b. Human Anatomy; c. Histology; d. Epidemiology and Biostatistics; and e. Social and Behavioral Sciences: Introduction to the Health Care System), Winter Quarter (a. Human Disease, consisting of Microbiology and Pathology sections; and b. Social and Behavioral Sciences: Psychopathology), and Spring Quarter (a. Human Disease, consisting of Microbiology and Pathology sections; and b. Lab Medicine).

Preclinical course grades in all years were determined primarily from examination results but in some cases included small contributions from laboratory and small group performance scores. Due to the subjective nature of laboratory and small group performance metrics, only the exam percentage scores were included in this data analysis. The implementation of the H/P/F grading, when applicable, used a fixed distribution of test scores to assign students into the honors group, such that honors assignments were restricted to a fixed percentage of students. Students from all years were subsequently graded with an H/P/F system during their clinical clerkships during the third and fourth years of medical school.

### Data collection

Student performance on preclinical (MS1 and MS2) courses was compiled and consisted of written and laboratory examination percent correct scores. There were a total of 31 separate examinations given during the MS1 year, and a total of 26 examinations in the MS2 year. In order to create a uniform sample of test scores across classes, examinations that were not available for all classes were excluded from the analysis. The consistent pool of exams included for analysis consists of 29 MS1 and 25 MS2 exams. In other words, every examination included in the analysis was administered to all students in all three classes. USMLE Step 1 scores were also obtained for all students in the sample. Finally, the following demographic variables were gathered: gender, age, in-state applicant status, MCAT biological sciences score, MCAT physical sciences score, MCAT verbal reasoning score, undergraduate science GPA, and undergraduate major. Table 1 provides a summary of these measures for the two groups of students. IRB approval was obtained before any data were collected or analyzed for this study.

**Table 1:** Demographic characteristics for members of the constant-grading classes\and the grading-change class

**Description**	**H/P/F grading**			**P/F grading**			**Total**			***t‒*** **test**		
	**Mean**		**SD**	**Mean**		**SD**	**Mean**		**SD**	***t‒*** **sta.s.c**		***p‒*** **value**
Number		243			117			360			-	
Sex (0 = Male, 1 = Female)	0.492	0.501	0.479	0.502	0.487	0.501	0.232	0.816	0.492	0.501	0.479	0.502
Age	23.34	3.10	22.94	2.76	23.21	2.99	1.192	0.234	23.34	3.10	22.94	2.76
In-state applicant (0 = No, 1 = Yes)	0.923	0.267	0.910	0.288	0.919	0.273	0.427	0.669	0.923	0.267	0.910	0.288
MCAT: Biological sciences	11.62	1.40	11.70	1.47	11.65	1.42	0.489	0.625	11.62	1.40	11.70	1.47
MCAT: Physical sciences	11.31	1.88	11.58	1.74	11.39	1.84	1.308	0.192	11.31	1.88	11.58	1.74
MCAT: Verbal	10.16	1.69	10.16	1.72	10.16	1.70	0.009	0.993	10.16	1.69	10.16	1.72
MCAT: Total	33.09	3.79	33.41	3.72	33.18	3.76	0.752	0.453	33.09	3.79	33.41	3.72
Medical school - First year exams	83.93	5.21	84.66	5.32	84.17	5.25	1.231	0.219	83.93	5.21	84.66	5.32
Medical school - Second year exams	82.28	5.17	81.41	4.93	82.00	5.10	1.518	0.130	82.28	5.17	81.41	4.93
USMLE - Step 1 score	229.95	18.86	231.72	18.01	230.54	18.57	0.844	0.399	229.95	18.86	231.72	18.01
Major: Biology	0.560	-	0.709	-	0.608	-	-	-	0.560	-	0.709	-
Major: Chemistry	0.165	-	0.145	-	0.158	-	-	-	0.165	-	0.145	-
Major: MECS	0.165	-	0.051	-	0.128	-	-	-	0.165	-	0.051	-
Major: Non-Science	0.074	-	0.085	-	0.078	-	-	-	0.074	-	0.085	-
Major: Missing	0.037	-	0.009	-	0.028	-	-	-	0.037	-	0.009	-

Notes and sources:

Medical school exams represent the average exam score across a common set of exams.

MECS stands for mathematical, engineering, & computer sciences.

Mapping from undergraduate major to undergraduate major category are provided in Additional file 2.

### Statistical analysis

To assess the effect of the grading policy change on academic performance of members of the class of 2013, the average percent correct score for the MS1 and MS2 years was computed for each student. Student performance during MS1 served as a baseline performance metric due to the unchanged grading policy in that year. A difference score was computed by comparing the overall performance of the students on examinations from their MS1 year compared to the MS2 year. As described above, students in the constant-grading classes (class of 2011 and 2012) were graded as H/P/F throughout the MS1 and MS2 years, while the grading-change class (class of 2013) was graded as H/P/F during the MS1 year, but P/F during the MS2 year.

Two-sample independent *t*-tests were used to compare the average performance during the MS1 and MS2 years for the constant-grading classes and grading-change class, in addition to the difference score that captured any decline in performance between the MS1 and MS2 years.

A linear regression analysis was conducted to determine which demographic characteristics (e.g., age, gender, MCAT performance, etc.) were associated with academic performance during medical school as well as to assess whether the grading policy change affected student performance after controlling for other factors that could be predictive of test scores. A second linear regression was conducted to assess the impact of the grading policy change as well as the impact of the demographic characteristics in predicting performance on the USMLE Step 1 examination. All statistical analyses were conducted using Stata version 12 (StataCorp LP, College Station, TX).

## Results

### Demographic characteristics

Demographic characteristics for members of the constant-grading and grading-change classes are presented in Table 1. The classes were found to be comparable and there were no statistically significant differences in gender, age, MCAT scores, undergraduate science GPA, undergraduate major, or in-state application status (for all comparisons, *p* > 0.10).

### The impact of grading policy on performance on preclinical coursework

**Figure 1:**
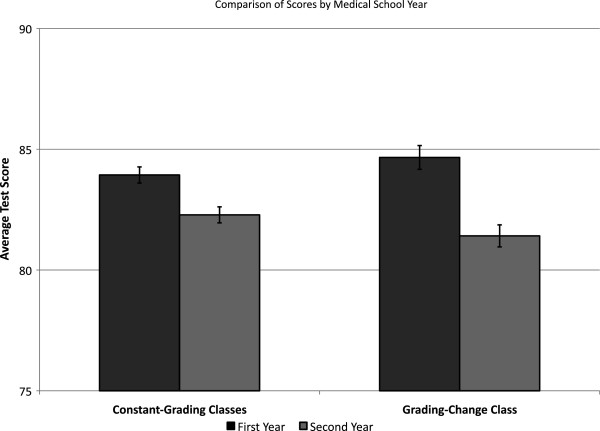
Average performance on first-year and second-year preclinical examinations for members of the constant-grading classes and grading-change class.

**Figure 2:**
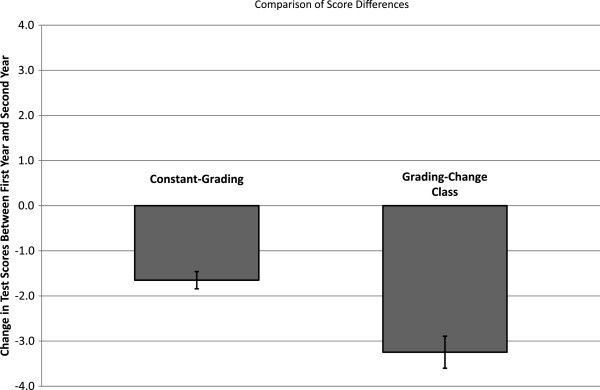
The difference score reflecting a change in average performance between MS1 and MS2 years for members of the constant-grading classes and grading-change class.

Average performance on first-year and second-year preclinical examinations is shown in Figure 1. The difference score reflecting a change in average performance between MS1 and MS2 years is presented in Figure 2 for the constant-grading and grading-change classes. There was a statistically significant difference in the difference scores as a function of grading policy change such that the constant-grading classes exhibited a 1.65% point decrease between the MS1 and MS2 academic years, whereas the grading-change class experienced a 3.25% point decrease in the MS2 year compared to the MS1 year (*t* = 4.32, *p* < 0.0001). In other words, the class that experienced a grading policy change to P/F during the MS2 year (grading-change class) exhibited a greater decline in overall performance relative to the classes who were graded H/P/F throughout the MS1 and MS2 years (constant-grading classes).

A multiple linear regression analysis was conducted to examine the relationship between performance on preclinical examinations and the following 10 potential predictors: Test Year (MS1 or MS2 coursework), Grading Policy (grading-change or constant-grading), Gender, Age, In-State Applicant Status, MCAT Biological Sciences, MCAT Physical Sciences, MCAT Verbal Reasoning, Undergraduate Science GPA, and Undergraduate Major. Performance on preclinical examinations was significantly predicted by the following factors: Grading Policy (Coefficient = 1.779), Test Year (Coefficient = -1.684), Grading Policy × Test Year (Coefficient = -1.539), MCAT Biological Sciences score (Coefficient = 1.240), MCAT Verbal Reasoning score (Coefficient = 0.532), and Undergraduate Science GPA (Coefficient = 6.114) and R^2^ = .194. See Column (6) of Table 2. Coefficients indicate the percentage point change in test scores resulting from a one-point change in the independent variable. Regressions on first-year scores and second-year scores only yielded, as expected, a significant impact of the grading change on second-year scores but not first-year scores. See Columns (2) and (4) of Table 2. All regressions utilize student fixed effects, robust standard errors, and clustering of standard errors by individual students.

**Table 2:** Multiple linear regression analysis examining the relationship between performance on preclinical examinations and the listed predictors

**Variable**	**First**	**Year**	**Scores**	**First**	**Year**	**Scores**	**Both**	**Year**	**Scores**
	**(1)**		**(2)**	**(3)**		**(4)**	**(5)**		**(6)**
P/F grading	0.728		0.350	-0.908*		-1.409***	2.331***		1.779**
	(0.513)		(0.491)	(0.473)		(0.444)	(0.800)		(0.843)
Test year (Year 1 = 0, Year 2 = 1)							-1.649***		-1.684***
							(0.192)		(0.200)
P/F grading × Test Year							-1.614***		-1.539***
							(0.404)		(0.469)
Sex (1 = female, 0 = male)			0.104			0.829			0.468
			(0.544)			(0.516)			(0.486)
Age			-0.0294			0.0327			0.00157
			(0.0945)			(0.0775)			(0.0782)
In-state applicant (1 = yes, 0 = no)			-0.0259			-1.714*			-0.869
			(1.058)			(0.913)			(0.914)
MCAT: Biological sciences			1.421***			1.060***			1.240***
			(0.230)			(0.222)			(0.209)
MCAT: Physical sciences			0.407**			0.194			0.301**
			(0.159)			(0.161)			(0.150)
MCAT: Verbal reasoning			0.302*			0.762***			0.532***
			(0.159)			(0.140)			(0.131)
Undergraduate GPA - Science			6.314***			5.913***			6.114***
			(1.073)			(1.034)			(0.961)
Major: Chemistry			0.288			1.278			0.784
			(0.833)			(0.777)			(0.734)
Major: MECS			-0.673			-1.332**			-1.003
			(0.748)			(0.613)			(0.619)
Major: Other			-2.548***			-0.859			-1.701*
			(0.903)			(1.013)			(0.872)
Constant		80.24***	34.51***	81.04***		38.42***	83.11***		38.99***
		(1.516)	(5.063)	(0.473)		(4.609)	(0.782)		(4.345)
Student fixed effects		Yes	Yes	Yes		Yes	Yes		Yes
Observations		8,633	7,289	8,622		7,287	17,255		14,576
R-squared		0.075	0.174	0.115		0.227	0.096		0.194

Notes and sources:

*p-value < 0.1, **p-value < 0.05, ***, *p*-value < 0.01.

Standard errors in parentheses represent robust standard errors with clustering by individual student.

“Major: Biology” has been referenced as the baseline major.

Regression results indicate that student performance on preclinical exams was positively associated with MCAT Biological Sciences, MCAT Verbal Reasoning, MCAT Physical Sciences, and Undergraduate Science GPA. The positive coefficient on the grading change indicates that students in the class of 2013 had a higher baseline score by 1.779 points than in the classes of 2011 and 2012, controlling for other factors. The negative coefficient on test year indicates that students had lower test scores, on average, on MS2 coursework compared to MS1 coursework by 1.684 points. Finally, the negative coefficient on the interaction between P/F grading and test year indicates that students with P/F grading in the second year performed relatively worse, on average, on MS2 clinical coursework compared to the constant-grading classes by 1.539 points. All of the above results are robust to omitting examination scores from the first quarter of the MS1 year, in which all students from all classes were graded P/F.

### USMLE step 1 performance

A multiple linear regression analysis was conducted to examine the relationship between USMLE Step 1 scores and the following 10 potential predictors: Grading Policy (grading-change or constant-grading), Score Difference (average performance in the MS2 year coursework – average performance in MS1 year coursework), Gender, Age, In-State Applicant Status, MCAT Biological Sciences, MCAT Physical Sciences, MCAT Verbal Reasoning, Undergraduate Science GPA, and Major. The analysis revealed that USMLE Step 1 scores were significantly predicted by the following factors (*p* < 0.05 or *p* < 0.01): Score Difference (Coefficient = 0.822), MCAT Biological Sciences score (Coefficient = 4.562), MCAT Verbal Reasoning score (Coefficient = 1.477), MCAT Physical Sciences score (Coefficient = 1.147), and Undergraduate GPA – Science (Coefficient = 11.13). There was a trend for the following factors to predict USMLE Step 1 performance (0.1 > *p* > 0.05): Gender (Coefficient = -3.389), and Age (Coefficient = -0.615). The negative coefficient on Gender indicates that there was a trend toward men scoring an average of 3.389 points higher than women on the USMLE Step 1; and the negative coefficient on Age indicates that for every year of advanced age, students scored an average of 0.621 points lower on the USMLE Step 1, although neither factor reached significance at the *p* < 0.05 level. The multiple regression model with all ten predictors produced R^2^ = .351. See Column (4) of Table 3.

**Table 3:** Multiple linear regression analysis examining the relationship between USMLE Step 1 scores and the listed predictors

**Variable**	**Dependent variable: USMLE step 1 score**			
	**(1)**	**(2)**	**(3)**	**(4)**
P/F grading		1.572		2.810
Score difference (Year 2 minus Year 1)			0.738***	0.822***
			(0.283)	(0.293)
Sex (1 = female, 0 = male)	-3.184*	-3.130*	-3.456*	-3.389*
	(1.769)	(1.773)	(1.765)	(1.767)
Age	-0.526	-0.519	-0.618*	-0.615*
	(0.323)	(0.321)	(0.331)	(0.329)
In-state applicant (1 = yes, 0 = no)	-2.764	-2.735	-2.565	-2.490
	(3.008)	(3.035)	(2.950)	(2.993)
MCAT: Biological sciences	4.274***	4.301***	4.489***	4.562***
	(0.818)	(0.822)	(0.805)	(0.812)
MCAT: Physical sciences	1.006*	0.967*	1.194**	1.147**
	(0.571)	(0.579)	(0.571)	(0.578)
MCAT: Verbal reasoning	1.731***	1.747***	1.477**	1.477**
	(0.594)	(0.595)	(0.606)	(0.607)
Undergraduate GPA - Science	11.59***	11.49***	11.34***	11.13***
	(4.272)	(4.288)	(4.260)	(4.279)
Major: Chemistry	3.684	3.822	3.138	3.324
	(2.456)	(2.441)	(2.538)	(2.514)
Major: MECS	3.431	-3.045	-3.279	-2.572
	(3.174)	(3.216)	(3.165)	(3.208)
Major: Other	1.619	-1.646	-2.478	-2.623
	(3.201)	(3.217)	(3.176)	(3.197)
Constant	125.1***	124.7***	127.7***	127.3***
	(18.51)	(18.39)	(18.74)	(18.62)
Observa_ons	298	298	298	298
R-squared	0.330	0.332	0.347	0.351

Notes and sources:

*p-value < 0.1, **p-value < 0.05, ***, *p*-value < 0.01.

Standard errors in parentheses represent robust standard errors with clustering by individual student.

“Major: Biology” has been referenced as the baseline major.

Regression results indicate that student performance on USMLE Step 1 was positively associated with MCAT Biological Sciences, MCAT Verbal Reasoning Score, MCAT Physical Sciences, and Undergraduate Science. The positive coefficient on the score difference indicates that students whose average performance on preclinical examinations increased during the MS2 year relative to the MS1 year performed better than students whose performance decreased in the MS2 year compared to the MS1 year, controlling for other factors in the model, by 0.822 USMLE Step 1 points per percentage point on preclinical score difference. This finding was observed for all students, regardless of grading policy, and was not significant when only the students from the grading-change class were included, perhaps due to insufficient power.

## Discussion

The issue of grading in medical school is important to both students and educators, and there is debate regarding the optimal method for grading of coursework during the preclinical years of medical school. On the one hand, several studies have shown the benefit of pass/fail grading in terms of improving student well-being [4,5], however the impact of such systems on academic outcomes is less clear. Whereas some studies have found no relationship between grading system and academic performance [4,7], other work has shown that numeric grades during the first year of medical school predict performance in subsequent years, performance on all 3 steps of the USMLE, as well as post-graduate ratings of clinical competence [2]. Limiting the debate is the relative lack of studies that investigate the effect of grading policy on medical student performance on preclinical coursework as well as performance on the USMLE Step 1 examination. The present study aimed to address this need by evaluating a unique natural experiment at the UC San Diego School of Medicine to assess the effect of pass/fail grading on preclinical performance and USMLE Step 1 scores.

Although this paper is not the only one to investigate how a change to pass/fail grading impacts performance in medical school, it is unique in that it addresses how a grading change affected a single cohort of students relative to similar cohorts that did not undergo a grading change. Given the timing of the implemented grading policy change at UCSD, we were able to evaluate how the same group of students performed when exposed to both grading systems – allowing for greater ability to detect any impact the grading policy change had on student performance. Other studies that have published findings based on separate cohorts of students exposed to separate grading policies do not have this added statistical advantage.

Our study found that a change from an Honors-Pass-Fail grading system to a Pass-Fail grading system was associated with a modest decrease in performance on preclinical coursework but with no effect on performance on the USMLE Step 1 examination. These findings persisted after controlling for other factors that are predictive of performance on medical school examinations and USMLE Step 1 performance (including undergraduate science GPA, and MCAT scores).

Although the grading policy, per se, did not have an impact on USMLE Step 1 performance, our study demonstrated an interesting relationship between performance on preclinical coursework and USMLE Step 1 scores. Some students in the grading-change class (the class of 2013) might have identified a potential “advantage” to the Pass/Fail grading system by prioritizing Step 1 preparation at the expense of MS2 coursework, given the lack of consideration for honors grading. However, this strategy was not supported by the data, which indicated that students, regardless of the grading policy, who performed differentially better in their MS2 year compared to their MS1 year scored better on the USMLE Step 1 examination.

A particular strength of the present investigation stems from the fact that the grading policy change occurred during the middle of the preclinical years for the class of 2013. As a result, performance of the same group of students could be assessed under both a P/F as well as an H/P/F grading system. This unique design afforded a greater ability to detect changes in performance associated with the grading policy as opposed to other factors. This may explain why our study found a significant impact of P/F grading on performance whereas other studies have not.

A potential limitation of the present study is that it does not address the psychological impact associated with these grading policies, the long-term impact of the pass/fail grading policy change on student performance on other USMLE Step exams, performance during the clinical years of medical school, performance in domains of skill rather than knowledge, or the impact of the grading system change on placement in residency programs, all of which are meaningful considerations for evaluating possible grading systems.

## Conclusion

In conclusion, our study provides an important demonstration that grading policy can impact student performance on medical school examinations. Students at the University of California, San Diego School of Medicine who were exposed to both H/P/F and P/F grading systems during their first 2 years of medical school performed significantly better on preclinical examinations under the H/P/F grading structure relative to two previous classes who were not exposed to such a grading policy change. However, we also showed that the same change in grading policy had no observable effect on USMLE Step 1 scores. Given the modest performance difference demonstrated in this study compared to known benefits of pass/fail grading on medical student psychological well-being (although well-being was not measured in this study) [4,5], it seems reasonable to recommend continuation of pass/fail grading in the future as a multitude of factors should be considered in assessing the merits of various grading systems.

### Ethical approval

The research project was certified as exempt from IRB approval by the University of California San Diego Human Research Protections Program. The exemption was granted on October 19, 2011 for the project number 11153XX.

## Abbreviations

GPA: Grade point average; USMLE: United states medical licensing examination; MCAT: Medical college aptitude test; UCSD: University of California, San Diego; MS1: first year of medical school; MS2: Second year of medical school; H/P/F: Honors/pass/fail; P/F: Pass/fail; IRB: Institutional review board; MECS: Math, engineering, and computer science.

## Competing interests

The authors have no financial or non-financial competing interests to declare.

## Authors’ contributions

SGRM conceived of the study, performed the statistical analysis, and drafted the manuscript. RDM performed statistical analysis, interpreted data, and provided intellectual guidance. JAF performed data collection, processing, and interpretation. CJK assisted in data interpretation and provided intellectual guidance. MCS assisted in data interpretation and provided intellectual guidance. JM helped conceive of the study, participated in coordination of data collection, helped to draft the manuscript, interpreted data, and provided intellectual guidance. All authors read and approved the final manuscript.

## Pre-publication history

The pre-publication history for this paper can be accessed here:

http://www.biomedcentral.com/1472-6920/14/127/prepub

## Supplementary Material

Additional file 1: Table S1 Summary of the grading policy used in each quarter for students in the constant-grading classes and the grading-change class. Click here for file

Additional file 2 Undergraduate major categories. Click here for file

## References

[B1] The AAMC OSR Preclinical Grading Questionnaire Resultshttps://www.aamc.org/download/185190/data/preclinical_grading.pdf

[B2] Gonnella JS Erdmann JB Hojat M An emprical study of the predictive validity of number grades in medical school using 3 decades of longitudinal data: implications for a grading system Med Educ 2004 38 425 434 10.1111/j.1365-2923.2004.01774.x 15025644

[B3] Spring L Robillard D Gehlbach L Simas TAM Impact of pass/fail grading on medical students’ well-being and academic outcomes Med Educ 2011 45 867 877 10.1111/j.1365-2923.2011.03989.x 21848714

[B4] Bloodgood RA Short JG Jackson JM Martindale JR A change to pass/fail grading in the first two years at one medical school results in improved psychological well-being Acad Med 2009 84 655 662 10.1097/ACM.0b013e31819f6d78 19704204

[B5] Reed DA Shanafelt TD Satele DW Power DV Eacker A Harper W Moutier C Durning S Massie S Thomas MR Sloan JA Dyrbye LN Relationship of pass/fail grading and curriculum structure with well-being among preclinical medical students: a multi-institutional study Acad Med 2011 86 1 7 10.1097/ACM.0b013e318205fe36 21952063

[B6] White CB Fantone JC Pass-fail grading: laying the foundation for self-regulated learning Adv in Health Sci Educ 2010 15 469 477 10.1007/s10459-009-9211-1 20012686

[B7] Robbins LS Fantone JC Oh MS Alexander GL Shlafer M Davis WK The effect of pass/fail grading and weekly quizzes on first-year students’ performances and satisfaction Acad Med 1995 70 327 329 10.1097/00001888-199504000-00019 7718068

