# Erratum: Evaluating a grading change at UCSD school of medicine: pass/fail grading is associated with decreased performance on preclinical exams but unchanged performance on USMLE step 1 scores

**DOI:** 10.1186/s12909-015-0341-2

**Published:** 2015-07-04

**Authors:** Susan GR McDuff, DeForest McDuff, Jennifer A Farace, Carolyn J Kelly, Maria C Savoia, Jess Mandel

**Affiliations:** 1 University of California, San Diego School of Medicine, 9500 Gilman Drive, San Diego, CA 92093 USA; 2 Quant Economics, Inc., San Diego, CA 92130 USA

## Erratum

After publication of [1], it emerged that a number of errors were introduced during typesetting affecting Table 1. The table can be viewed here as the authors originally intended.

**Table 1:** **Demographic characteristics**

Description	H/P/F Grading			P/F Grading			Total			*t‐* test		
	Mean		SD	Mean		SD	Mean		SD	*t‐* sta.s.c		*p--‐* value
Number		243			117			360			-	
Sex (0 = Male, 1 = Female)	0.492		0.501	0.479		0.502	0.487		0.501	0.232		0.816
Age	23.34		3.10	22.94		2.76	23.21		2.99	1.192		0.234
In-state applicant (0 = No, 1 = Yes)	0.923		0.267	0.910		0.288	0.919		0.273	0.427		0.669
MCAT: Biological sciences	11.62		1.40	11.70		1.47	11.65		1.42	0.489		0.625
MCAT: Physical sciences	11.31		1.88	11.58		1.74	11.39		1.84	1.308		0.192
MCAT: Verbal	10.16		1.69	10.16		1.72	10.16		1.70	0.009		0.993
MCAT: Total	33.09		3.79	33.41		3.72	33.18		3.76	0.752		0.453
Medical school - First year exams	83.93		5.21	84.66		5.32	84.17		5.25	1.231		0.219
Medical school - Second year exams	82.28		5.17	81.41		4.93	82.00		5.10	1.518		0.130
USMLE - Step 1 score	229.95		18.86	231.72		18.01	230.54		18.57	0.844		0.399
Major: Biology	0.560		-	0.709		-	0.608		-	-		-
Major: Chemistry	0.165		-	0.145		-	0.158		-	-		-
Major: MECS	0.165		-	0.051		-	0.128		-	-		-
Major: Non-Science	0.074		-	0.085		-	0.078		-	-		-
Major: Missing	0.037		-	0.009		-	0.028		-	-		-

*Notes and sources:*

Medical school exams represent the average exam score across a common set of exams.

MECS stands for mathematical, engineering, & computer sciences.

Mapping from undergraduate major to undergraduate major category are provided in Appendix 1.
