# Supplementary material for: Evaluating a grading change at UCSD school of medicine: pass/fail grading is associated with decreased performance on preclinical exams but unchanged performance on USMLE step 1 scores
Source: BMC Med Educ. 2014 Jun 30;14:127. doi: 10.1186/1472-6920-14-127 (PMC4083104; doi:10.1186/1472-6920-14-127)
Supplement: Additional file 1: Table S1 — Summary of the grading policy used in each quarter for students in the constant-grading classes and the grading-change class. [file 1472-6920-14-127-S1.pdf]

Supplemental Table A

Overview of Grading Systems

Class	First Year			Second Year		
	Fall	Winter	Spring	Fall	Winter	Spring
Constant-Grading Classes	P/F	H/P/F	H/P/F	H/P/F	H/P/F	H/P/F
Grading-Change Class	P/F	H/P/F	H/P/F	P/F	P/F	P/F

Notes and sources:
"P/F" stands for Pass/Fail.
"H/P/F" stands for High Pass/Pass/Fail.
Shaded cells represent the policy change to Pass/Fail for second year grading.
