# Supplementary material for: Evaluating a grading change at UCSD school of medicine: pass/fail grading is associated with decreased performance on preclinical exams but unchanged performance on USMLE step 1 scores
Source: BMC Med Educ. 2014 Jun 30;14:127. doi: 10.1186/1472-6920-14-127 (PMC4083104; doi:10.1186/1472-6920-14-127)
Supplement: Additional file 2 — Undergraduate major categories. [file 1472-6920-14-127-S2.pdf]

Appendix 1

Undergraduate Major Categories

Undergraduate Major	Category
American Studies & History	Non-Science
Animal Physio & Neuro.	Biology
Animal Physio & Neuro., Psych.	Biology
Animal Psych & Neuroscience/ Anthro	Biology
Anthropology	Non-Science
Anthropology/Molecular Bio	Biology
Biochem	Chemistry
Biochem & Cell Bio	Chemistry
Biochem & Chemistry	Chemistry
Biochem & Molecular Bio	Chemistry
Biochem & Molecular Biology	Chemistry
Biochem & Neurobiology	Chemistry
Biochem and Cell Bio	Chemistry
Biochem and Psych	Chemistry
Biochem/Economics	Chemistry
Biochem/Molecular & Cell Bio	Chemistry
Biochemistry	Chemistry
Biochemistry & Cell Bio	Chemistry
Biochemistry & Cell Bio/Psychology	Chemistry
Biochemistry & Molecular Bio	Chemistry
Biochemistry/Chemistry	Chemistry
Biochemistry/Psychology	Chemistry
Biochemisty & Cell Bio	Chemistry
Bioengineering	MECS
Bioengineering & Material Science Engineering	MECS
Bioengineering Tech./Bio Services	MECS
Bioengineering/Biotech	MECS
Bioengineering-Premed	MECS
Biol Anthropology and Anatomy/Epidemiology of M	Non-Science
Biological Basis of Behavior	Biology
Biological Basis of Behavior, Bio	Biology
Biological Psychology	Biology
Biological Sciences	Biology
Biology	Biology
Biology & Chemistry	Biology
Biology & Psychology	Biology
Biology/Cog Sci	Biology
Biomedical Engineering	MECS
Biomedical Engineering, Premed	MECS
Biomedical Engineering/Electrical Engineering	MECS
Biophysics	MECS

Undergraduate Major	Category
Biopsychological	Biology
Biopsychology	Biology
Biotechnology	MECS
Business Admin & Computer Science	MECS
Business Admin/Molecular and Cell Bio	Biology
Business Administration	Non-Science
Cell Bio	Biology
Chemical Bio	Chemistry
Chemical Biological Engineering	Chemistry
Chemical Biology	Chemistry
Chemical Engineering	MECS
Chemical Engineering/Biochemistry	Chemistry
Chemistry	Chemistry
Chemistry & Biochem	Chemistry
Chemistry, Biology	Chemistry
Chemistry/Immunology	Chemistry
Cognitive Science	Biology
Cognitive Science/Italian	Biology
Comparative Lit	Non-Science
Comparative Lit/Biology	Biology
Comparative Study of Religion	Non-Science
Computer Sci: bioinformatics	MECS
Computer Science	MECS
Computer Science: Bioinformatics	MECS
Cybermetics/Biochem and Molecular Bio	Chemistry
Earth Sciences	Biology
Economics	MECS
Electircal Engineering	MECS
Electircal Engineering/Computer Science, Applied Pl	MECS
Electrical Engineering	MECS
Electrical Engineering & Computer Science	MECS
Electrical Engineering and Physics	MECS
Engineering & Applied Science	MECS
English	Non-Science
English, Amer. Lit and Language	Non-Science
English/Biochem & Molecular Bio	Biology
English/Biological Sciences	Biology
Evolution & Ecology	Biology
Exercise Biology	Biology
Food Science and Tech	Non-Science
Genetics	Biology
History	Non-Science
Human Bio	Biology
Human Bio/Psychology	Biology

Undergraduate Major	Category
Human Biology	Biology
Human Biology/Psychology	Biology
Human Development	Biology
Humanities	Non-Science
Integrative Bio	Biology
International Relations	Non-Science
International Studies	Non-Science
Kinesiology	Non-Science
Life Science	MECS
Marine Biology	MECS
Material Science & Engineering	MECS
Material Science and Engineering	MECS
Mathmatics	MECS
Mathmatics & Systems Engineering	MECS
Microbial Biology	Biology
Microbio., Immun. & Mol. Gen.	Biology
Microbiology and Bacteriology	Biology
Microbiology, Immunology & Molecular Genetics	Biology
Mol. Cell and Dev. Bio	Biology
Molec. Cell. & Devel. Bio	Biology
Molecular & Cell Bio	Biology
Molecular & Cell Bio, Cellular & Dev. Bio, Econ	Biology
Molecular & Cell Bio/Neurobio	Biology
Molecular & Environmental Bio	Biology
Molecular and Cell Bio	Biology
Molecular Bio	Biology
Molecular Bio & Biochem	Biology
Molecular Bio & Microbio/Clinical Research	Biology
Molecular Biology	Biology
Molecular Biophysics & Biochemistry	Biology
Molecular Cell Bio	Biology
Molecular Cellular & Dev. Bio	Biology
Molecular Cellular & Developmental Bio	Biology
Molecular, Cell & Dev. Bio	Biology
Molecular, Cell and Develop. Bio	Biology
Music	Non-Science
Music/Molecular & Cell Bio	Biology
Neurobiology	Biology
Neurobiology,Physiology & behavior	Biology
Neuroscience	Biology
Neuroscience & Behavioral Bio	Biology
Neuroscience/Biology	Biology
Nuerobio, Physiology & Behavior	Biology
Pharmacology	Biology

Undergraduate Major	Category
Philosophy	Non-Science
Physics	MECS
Physiological Sciences	Biology
Physiology	Biology
Physiology/Neuroscience	Biology
Political Economy	Non-Science
Political Science	Non-Science
Pre-med/Psych	Biology
Psychobiology	Biology
Psychology	Biology
Psychology & Neuroscience	Biology
Psychology/English/Microbiology and Bacteriology	Biology
Psychology/Neurobiology, Physiology & Behavior	Biology
Psychology/Neuroscience	Biology
Social Science	Biology
Sociology	Non-Science
Spanish	Non-Science
Spanish/Bio	Biology
Spanish/Molecular & Cell Bio	Biology
Symbolic Systems	MECS
Zoology	Biology

Notes and sources:
MECS stands for mathematical, engineering, & computer sciences.
